# Predictors of survival in elderly patients undergoing surgery for glioblastoma

**DOI:** 10.1093/noajnl/vdab083

**Published:** 2021-06-21

**Authors:** Mathew R Voisin, Sanskriti Sasikumar, Gelareh Zadeh

**Affiliations:** 1 Division of Neurosurgery, Department of Surgery, University of Toronto, Toronto, Ontario, Canada; 2 Division of Neurology, Department of Medicine, University of Toronto, Toronto, Ontario, Canada

**Keywords:** comorbidities, complications, elderly, glioblastoma (GBM), outcomes

## Abstract

**Background:**

Glioblastoma (GBM) has a median age of diagnosis of 64 years old and the incidence increases with age. An increasing number of elderly patients are being diagnosed with GBM and undergoing surgery. These patients often present with multiple medical comorbidities and have significantly worse outcomes compared to adult patients. The goal of this study was to determine clinical predictors of survival in elderly patients undergoing surgery for GBM.

**Methods:**

Our brain tumor database was reviewed for all patients 65 years of age and older that underwent surgery for newly diagnosed GBM over a 14-year period from 2005 to 2018. Patient characteristics, comorbidities, complications, and treatment were collected. A total of 150 patients were included, and subdivided into two age categories; 65–74 years old and 75 years or older.

**Results:**

The median OS for all patients was 9.4 months. Neither the presence nor number of medical comorbidities were associated with decreased survival (*P* = .9 and *P* = .1, respectively). Postoperative complications were associated with worse survival for all patients (HR = 2.34, *P* = .01) and occurred in patients in the older age category and patients with longer lengths of stay (*P* < .0001).

**Conclusions:**

The presence of medical comorbidities is not a reason to exclude patients with GBM from surgical consideration. Excluding EOR and adjuvant treatment, postoperative complication is the most significant predictor of survival in elderly patients. Postoperative complications are associated with a longer LOS and are more common in patients 75 years of age and older.

**Key Points**

- Medical comorbidities are not associated with decreased survival in elderly patients with GBM.
- Excluding EOR and adjuvant treatment, postoperative complication is the most significant factor associated with poor survival in elderly patients with GBM.

**Importance of the Study**

As our population continues to age, an increasing number of elderly patients are being diagnosed with GBM and undergoing surgery, however, there still exists a major gap in the literature regarding the best management for these patients during the perioperative period. We conducted a single-center retrospective review on elderly patients (≥65 years old) undergoing surgery for primary GBM over a 14-year period. The presence of medical comorbidities was not associated with decreased survival and is not a reason to exclude elderly patients from surgery for GBM. Postoperative complication was the most significant factor related to poor outcome during hospital admission. Postoperative complications occurred in patients 75 years of age and older, and in patients with a longer LOS.

Glioblastoma (GBM) is the most common and deadly primary brain tumor with a median overall survival (OS) of less than 15 months despite current treatment.^1,2^ GBM has a median age of diagnosis of 64 years old and the incidence increases with age, peaking at 75–84 years old.^2^ Despite the high incidence of GBM in the elderly population, there has been only a paucity of studies examining GBM treatment and outcomes in elderly patients. The Stupp trial in 2005 that formed the current standard of care for primary GBM, including maximal safe surgical resection, followed by adjuvant radiation and temozolomide (TMZ) excluded all patients >70 years of age.^3^ Elderly patients often have more medical comorbidities, worse functional status, and more complications than nonelderly patients newly diagnosed with GBM.^4,5^ Outcomes in elderly patients with GBM are also significantly worse than nonelderly patients, with overall survival ranging from 6 to 12 months, depending on adjuvant treatment regimens.^5^

Traditionally, neurosurgeons have been hesitant to offer surgical management to these patients due to perceived lower surgical tolerance and poor longevity, and when offered, was often in the form of a biopsy for tissue diagnosis rather than an attempt at gross total resection.^5^ Historically, these patients were often treated with radiotherapy alone or more recently in those with O_6_-methylguanine-DNA methyl-transferase (*MGMT*) gene promoter methylation, TMZ.^5^ A recent systematic review and meta-analysis on elderly patients with GBM found increased extent of resection in elderly patients was associated with longer survival with no difference in morbidity and mortality.^6^ Two recent randomized controlled trials for elderly patients – Perry et al. and the Nordic group demonstrated increased survival in elderly patients with glioblastoma that were treated with a shortened course of radiotherapy in combination with TMZ compared to a standard course of radiotherapy or radiotherapy alone.^7,8^

Today, an increasing number of elderly patients with GBM are undergoing surgery. This is in part due to the increasing incidence of GBM overall, and the increasing incidence of GBM especially in the elderly.^9^ Furthermore, both the mean age of patients undergoing surgical intervention and the number of patients aged 75 and older undergoing surgery have significantly increased in the last 15 years.^10^ As the number of elderly patients with GBM undergoing surgery continues to increase, we need to determine how best to deliver perioperative care to improve outcomes and minimize complications in these patients.

We sought to determine clinical predictors of survival in elderly patients with newly diagnosed GBM undergoing surgical resection in order to identify high-risk groups and improve outcomes in this vulnerable population.

## Materials and Methods

### Patient Selection

Our hospital brain tumor biobank database was searched for all patients over a 14-year period, from January 1, 2005 to December 31, 2018. This database includes all patients undergoing surgery for a central nervous system (CNS) tumor that was collected at the time of surgery. All patients 65 years of age or older with a pathological diagnosis of de novo primary GBM were included. This database includes all patients/caregivers that consented to have their deidentified information used for potential future research purposes. All demographic, surgical, medical, and hospital admission history was collected retrospectively through chart review. No specific surgical selection criteria were used, and a total of 12 neurosurgeons from our institution performed the surgeries. Institutional review board (IRB) approval was obtained prior to study commencement. Patient consent was not required as this was a retrospective chart review.

### Patient Variables

Date of diagnosis was defined as the date of first imaging demonstrating an intracranial lesion. Overall survival information was calculated from the date of diagnosis to date of death or date of last follow-up. Karnofsky performance score (KPS) was recorded and if not explicitly mentioned determined retrospectively based upon clinical and operative notes. Extent of resection (EOR) was determined by the operating notes with gross total resection (GTR) defined as >90% resection and subtotal resection (STR) as <90% resection, or biopsy. All patient medical records and discharge summaries were reviewed and any complication or deviation from a routine discharge home was recorded.

### Statistical Analysis

Descriptive statistics were determined for all patient characteristics. Tables 1 and 2 include analysis of continuous and categorical variables using t-tests and chi-squared tests, respectively. Overall chi-square tests with pairwise analyses were done with the use of Fisher's exact test if absolute values were less than 5. A nonparametric Kaplan–Meier survival analysis and Cox proportional hazards model were used for overall survival with other variables. A logistic regression model was used for postoperative complications. Tables 3 and 4 were completed using univariate and multivariate analyses. All variables in the univariate analyses with *P*-values < .05 were included in the multivariate models. *P*-values of <.05 were considered significant. All statistical analyses and figures were completed using R v3.6.2.^11^

**Table 1.** Patient Baseline Characteristics and Comorbidities

Characteristic *n* (%)	Cohort			*P*-value	
	All	Age 65–74	Age 75+		
Total # of patients	150	79	71	.42	
Age at diagnosis in years					
Mean	74.5	69.4	80.1	**<.0001**	
Median	74	70	79		
Range	65–94	65–74	75–94		
Sex					
Males	79 (52.7)	43 (54.4)	36 (50.7)	.77	
Females	71 (47.3)	36 (45.6)	35 (49.3)		
Preop KPS					
Mean	77.5	76.6	78.6	.37	
Median	80	80	80		
Range	10–100	10–100	50–100		
Comorbidities					
Hypertension	90 (60)	43 (54.4)	47 (66.2)	**.04**	
Diabetes	30 (20)	**10 (12.7)**	**20 (28.2)**		**.03**
Dyslipidemia	53 (35.3)	30 (38)	23 (32.4)		
Atrial fibrillation	10 (6.7)	3 (3.8)	7 (9.9)		
Cardiovascular disease	29 (19.3)	11 (13.9)	18 (25.4)		
COPD	7 (4.7)	3 (3.8)	4 (5.6)		
Smoker	21 (14)	**16 (20.3)**	**5 (7)**		**.04**
Other CNS disease	27 (18)	17 (21.5)	10 (14.1)		
Other cancer	34 (22.7)	14 (17.7)	20 (28.2)		
Mean # of comorbidities	2	1.8	2.2	.18	
# With any comorbidities	124 (82.7)	64 (81)	60 (84.5)	.73	
# With multiple comorbidities	82 (54.7)	39 (49.4)	43 (60.6)	.22	

Significant values (*P*-value < 0.05) are given in bold.

**Table 2.** Patient Treatment and Course in Hospital

Characteristic *n* (%)	Cohort			*P*-value	
	All	Age 65–74	Age 75+		
Total # of patients	150	79	71	.42	
Overall survival (months)					
Median (95% CI)	9.4 (7.8–12.2)	10.8 (8.4–14.1)	7.2 (5.1–11.4)	.06	
EOR					
Biopsy	18 (12)	8 (10.1)	10 (14.1)	.7	
STR	104 (69.3)	55 (69.6)	49 (69)		
GTR	28 (18.7)	16 (20.3)	12 (16.9)		
Length of stay in days					
Mean	10.6	10.5	10.6	.98	
Median	5	4.5	6		
Range	0–107	0–90	0–107		
Discharge destination					
Home	94 (62.7)	56 (70.9)	38 (53.5)	.26	
Home with supports	9 (6)	4 (5.1)	5 (7)		
Inpatient rehab	24 (16)	10 (12.7)	14 (19.7)		
Palliative	19 (12.7)	8 (10.1)	11 (15.5)		
Death in hospital	4 (2.7)	1 (1.3)	3 (4.2)		
Adjuvant treatment					
Radiation	102 (68)	**69 (87.3)**	**33 (46.5)**	**<.0001**	**<.0001**
TMZ	75 (50)	**55 (69.6)**	**20 (28.2)**		**<.0001**
Chemoradiation	70 (46.7)	**55 (69.6)**	**15 (21.1)**		**<.0001**
None	42 (28)	**10 (12.7)**	**32 (45.1)**		**<.0001**
Postoperative complications					
UTI	15 (26.3)	7 (31.8)	8 (22.9)	.34	
Pneumonia	3 (5.3)	2 (9.1)	1 (2.9)		
C. difficile	1 (1.8)	0	1 (2.9)		
Sacral wound	1 (1.8)	1 (4.5)	0		
DVT	4 (7)	3 (13.6)	1 (2.9)		
PE	2 (3.5)	1 (4.5)	1 (2.9)		
CHF	2 (3.5)	0	2 (5.7)		
Delirium	17 (29.8)	4 (18.2)	13 (37.1)		
Poor mobility/frailty	12 (21.1)	4 (18.2)	8 (22.9)		
Surgical complications	6 (4)	1 (16.7)	5 (83.3)	.1	
Patients with one complication	45 (30)	**17 (21.5)**	**28 (39.4)**	**.03**	
Patients with multiple complications	15 (10)	6 (8.9)	8 (11.3)	.63	
Total infection-related complications	21 (33.3)	10 (43.5)	11 (27.5)	.52	
Total number of complications	63 (100)	**23 (100)**	**40 (100)**	**.0007**	

Significant values (*P*-value < 0.05) are given in bold.

**Table 3.** Clinical Predictors of Overall Survival

Characteristic	Univariate Cox		Multivariate Cox	
	HR (95% CI)	*P*-value	HR (95% CI)	*P*-value
Age	1.01 (0.97–10.44)	.61		
Sex	0.88 (0.56–1.36)	.56		
Medical comorbidities				
HTN	1.01 (0.66–1.55)	.96		
T2DM	**2.02 (1.22–3.34)**	**.01**	1.44 (0.81–2.56)	.21
Dyslipidemia	0.81 (0.52–1.27)	.36		
Smoking	1.28 (0.71–2.28)	.42		
Afib	1.04 (0.48–2.26)	.92		
CVD	1.39 (0.82–2.34)	.24		
COPD	1.0 (0.40–2.49)	.99		
Other CNS disease	1.39 (0.79–2.44)	.27		
Previous history of cancer	1.48 (0.88–2.49)	.16		
Number of comorbidities	1.14 (0.98–1.32)	.09		
Multiple comorbidities	0.98 (0.64–1.50)	.94		
Any comorbidity	1.05 (0.59–1.87)	.87		
Hospital stay and treatment				
Preop KPS	0.99 (0.97–1.0)	.14		
Postop complication	**2.83 (1.77–4.53)**	**<.0001**	**2.34 (1.19–4.6)**	**.01**
Length of stay	**0.46 (0.29–0.72)**	**.001**	**0.98 (0.97–0.99)**	**.03**
Discharge home	**0.50 (0.31–0.80)**	**.01**	0.54 (0.27–1.05)	.07
EOR		**.003**		
Biopsy	Reference			
STR	**0.33 (0.13–0.81)**	**.02**	**0.38 (0.15–0.98)**	**.046**
GTR	**0.17 (0.06–0.48)**	**.0007**	**0.24 (0.08–0.72)**	**.01**
Adjuvant treatment		**<.0001**		
None	Reference			
Radiation only	**0.27 (0.13–0.56)**	**.0005**	**0.37 (0.16–0.84)**	**.02**
TMZ only	**0.16 (0.04–0.57)**	**.005**	**0.08 (0.02–0.33)**	**.0005**
Radiation + TMZ	**0.09 (0.04–0.19)**	**<.0001**	**0.14 (0.06–0.33)**	**<.0001**

Significant values (*P*-value < 0.05) are given in bold.

**Table 4.** Clinical Predictors of Postoperative Complications

Characteristic	Univariate Logistic Regression		Multivariate Logistic Regression	
	OR (95% CI)	*P*-value	OR (95% CI)	*P*-value
Age	**1.02 (1.01–1.03)**	**.01**	**1.01 (1.00–1.02)**	**.006**
Sex	0.98 (0.85–1.14)	.8		
Medical comorbidities				
HTN	1.12 (0.96–1.30)	.15		
T2DM	**1.34 (1.12–1.60)**	**.002**	1.13 (0.96–1.34)	.15
Dyslipidemia	1.13 (0.97–1.31)	.13		
Smoking	0.98 (0.80–1.22)	.88		
Afib	0.90 (0.67–1.21)	.48		
CVD	**1.25 (1.04–1.51)**	**.02**	1.09 (0.91–1.30)	.37
COPD	0.99 (0.69–1.40)	.93		
Other CNS disease	0.95 (0.79–1.15)	.61		
Previous history of cancer	1.07 (0.90–1.28)	.76		
Number of comorbidities	**1.06 (1.01–1.11)**	**.02**	1.0 (0.95–1.06)	.99
Multiple comorbidities	1.10 (0.95–1.27)	.23		
Any comorbidity	1.09 (0.90–1.32)	.4		
Hospital stay and treatment				
Preop KPS	0.99 (0.99–1.0)	.24		
Length of stay	**1.02 (1.01–1.02)**	**<.0001**	**1.01 (1.01–1.02)**	**<.0001**
Discharge home	**0.63 (0.56–0.72)**	**<.0001**	**0.80 (0.70–0.91)**	**.001**
EOR				
Biopsy	Reference			
STR	1.0 (0.8–1.3)	.98		
GTR	0.83 (0.63–1.08)	.17		

Significant values (*P*-value < 0.05) are given in bold.

## Results

### Patient Characteristics and Comorbidities

A total of 150 patients aged ≥65 that underwent surgery for primary GBM were included in the study. Table 1 lists the patient baseline characteristics and comorbidities. A total of 124 patients (82.7%) had at least one medical comorbidity and 82 patients (54.7%) had multiple comorbidities. Between the two age groups, there was no difference between the number of patients with comorbidities, or the number of patients with multiple comorbidities (*P*-values = 0.73 and 0.22, respectively).

### Treatment and Course in Hospital

**Figure 1.**
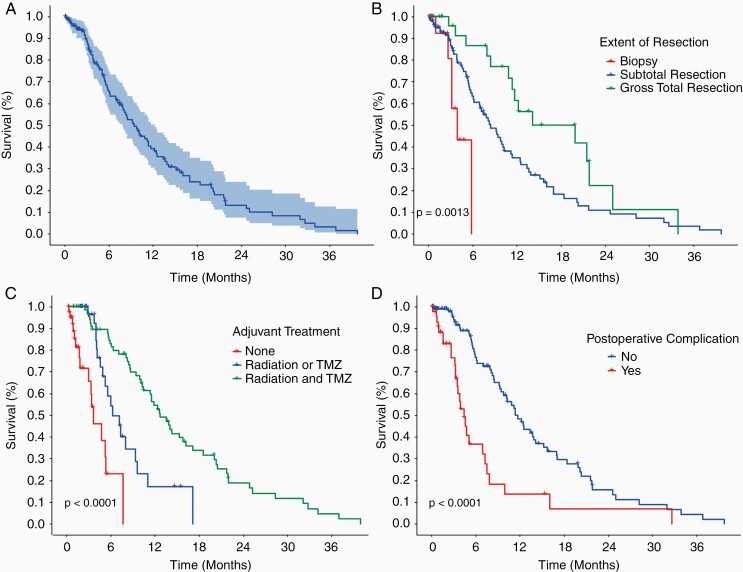
Survival curves for elderly patients undergoing surgery for GBM. (A) OS for all 150 patients with 95% CI. Median OS of 9.4 months (95% CI 7.8–12.2 months). (B) OS by extent of resection. Overall *P*-value = .0013. Biopsy versus STR (*P*-value = .02, HR = 0.33, 0.13–0.81), biopsy versus GTR (*P*-value < .001, HR = 0.17, 0.06–0.47), and STR versus GTR (*P*-value = .02, HR = 0.52, 0.3–0.92) were all associated with significantly longer survival. (C) OS by adjuvant treatment. Overall *P*-value < .0001. None versus radiation or TMZ (*P*-value = .0001, HR = 0.24, 0.12–0.5), None versus radiation and TMZ (*P*-value < .0001, HR = 0.09, 0.05–0.19), and radiation or TMZ versus radiation and TMZ (*P*-value < .001, HR = 0.38, 0.21–0.67) were all associated with significantly longer survival. (D) OS for patients with a postoperative complication was significantly less than those without a postoperative complication (*P*-value < .0001, HR = 2.8, 1.77–4.53). Median OS in patients without a postoperative complication was 11.4 months (9.7–15.2) and with a postoperative complication was 4.4 months (3.5–7.5).

Table 2 lists the patient course in hospital and adjuvant treatment regimen. The median OS for all patients was 9.4 months with a 95% CI of 7.8–12.2 months (Figure 1A). The median length of stay (LOS) was 5 days (range of 0–107) and the majority of patients were discharged home or home with supports (68.7%). A total of 45 patients (30%) had at least one postoperative complication, and 15 patients (10%) had multiple postoperative complications.

**Figure 2.**
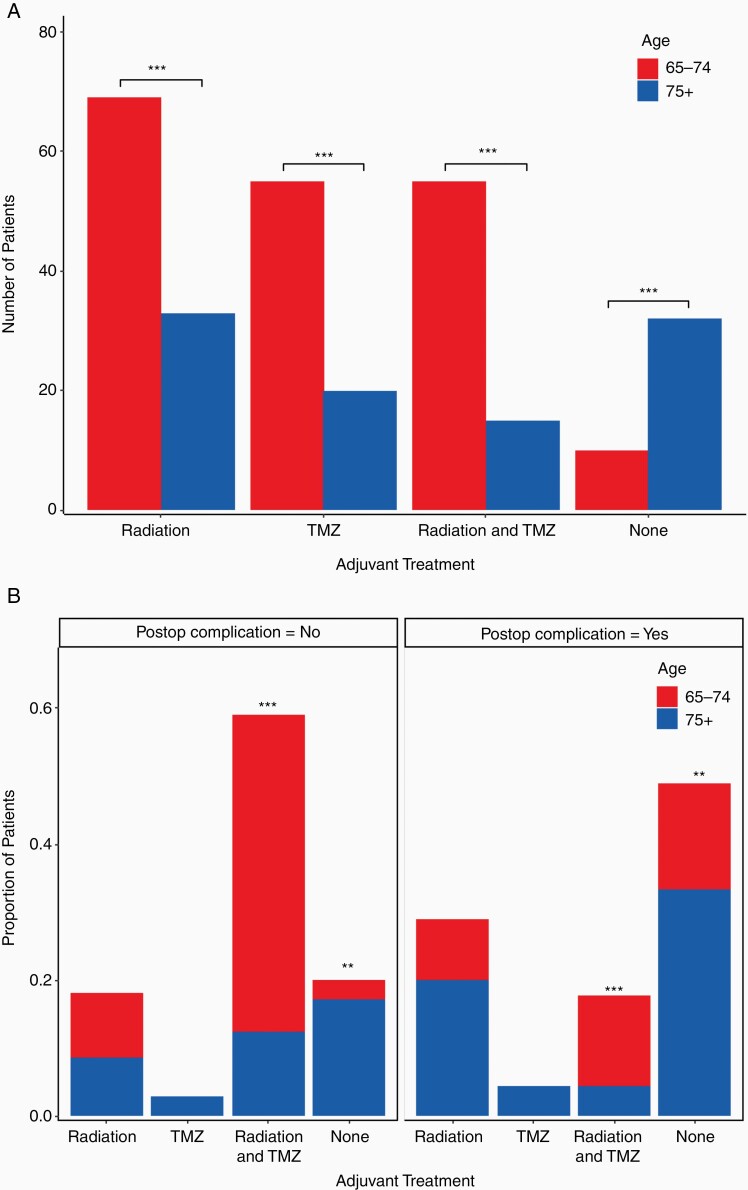
Adjuvant treatment by age and postoperative complication. (A) Postoperative adjuvant treatment subdivided by age 65–74 and 75+ for all 150 patients. Patients aged 65–74 were more likely to receive radiation, TMZ, or radiation and TMZ, and less likely to receive no adjuvant treatment than patients aged 75+ (all *P*-values < .0001 (***)). (B) Adjuvant treatment subdivided by postoperative complication and age. Patients from both age categories were more likely to receive radiation and TMZ and less likely to receive no adjuvant treatment if they did not have a postoperative complication (left panel vs right panel, *P*-values < .0001 (***) and < .001 (**), respectively). Patients aged 75+ were less likely to receive radiation and TMZ and more likely to receive no adjuvant treatment independent of postoperative complication (blue bars vs red bars, *P*-values < .0001 (***) and < .001 (**), respectively).

In our analysis, overall survival was not significantly different between elderly patients aged 65–74 and patients aged 75 years of age and older (10.8 vs 7.2 months, *P*-value = .07). However, adjuvant treatment is a confounding factor on age and overall survival, because patients aged 65–74 were more likely to receive adjuvant treatment of radiation, TMZ, or both, and less likely to receive no additional treatment than patients aged 75 years of age or older (Figure 2A, *P*-values < .0001 for all). Patients aged 75 years of age or older had a significantly higher total number of postoperative complications (*P*-value = .0007) and had a significantly higher number of patients with one postoperative complication (*P*-value = .03).

### Clinical Predictors of Overall Survival

All of the variables from Tables 1 and 2 were analyzed using a Cox proportional hazards model to determine their effects on OS, as represented in Table 3. Significant factors in the univariate analysis were added to a multivariate model. In keeping with previous studies, both EOR and adjuvant treatment were significantly related to improved OS in the multivariate model. Compared to biopsy alone, STR had a hazard ratio (HR) of 0.38 (*P*-value = .046, 95% CI 0.15–0.98), and GTR had a HR of 0.24 (*P*-value = .01, 95% CI 0.08–0.72). Adjuvant treatment with radiation alone (*P*-value = .02, HR = 0.37, 95% CI 0.16–0.84), TMZ alone (*P*-value = 0.0005, HR = 0.08, 95% CI 0.02–0.33), and combined radiation and TMZ (*P*-value < 0.0001, HR = 0.14, 95% CI 0.06–0.33) all resulted in increased survival in elderly patients with GBM. Figure 1B and C show Kaplan–Meier survival curves for OS versus EOR (*P*-value = .0013 overall) and adjuvant treatment (*P*-value < .0001), respectively. Neither the presence of any medical comorbidity, the number of comorbidities, nor the presence of multiple comorbidities were associated with decreased survival (*P*-value > .05 for all).

In addition to EOR and adjuvant treatment, a shorter length of stay (*P*-value = .03, HR = 0.98, 95% CI 0.97–0.99) was significantly related to improved survival and the presence of a postoperative complication (*P*-value = .01, HR = 2.34, 95% CI 1.19–4.6) was significantly related to decreased survival in the multivariate model. Figure 1D shows the Kaplan–Meier survival curve for OS versus postoperative complication (*P*-value < .0001).

### Clinical Predictors of Postoperative Complications

A logistic regression model was completed to determine significant clinical predictors of postoperative complications (Table 4). In the multivariate model, three factors were found to be significantly associated with elderly patients that had a postoperative complication. Both older age (*P*-value = .006, OR = 1.01, 95% CI 1–1.02) and longer LOS (*P*-value < .0001, OR = 1.01, 95% CI 1.01–1.02) were associated with increased risk, while discharge home (*P*-value = .001, OR = 0.8, 95% CI 0.7–0.91) was associated with decreased risk. The median LOS for patients without a postoperative complication was 4 days, compared to a median LOS of 17 days for patients that suffered a postoperative complication.

Finally, adjuvant treatment was subdivided by the presence or absence of a postoperative complication and also by age group as shown in Figure 2B. Patients that did not have a postoperative complication were more likely to receive combined adjuvant treatment with radiation and TMZ, compared with patients that suffered a postoperative complication (*P*-value < .0001). Conversely, patients that suffered a postoperative complication were more likely to receive no adjuvant treatment compared to those patients that did not suffer a postoperative complication (*P*-value < .001).

In patients that suffered a postoperative complication, age was not significantly related to receiving a different adjuvant treatment regimen (*P*-value = .09). In patients that did not suffer a postoperative complication, age was significantly related to receiving a different adjuvant treatment regimen (*P*-value < .0001). Patients aged 75 and older that did not suffer a postoperative complication were less likely to receive combined radiation and TMZ treatment and more likely to receive no adjuvant treatment than patients aged 65–74 that did not suffer a postoperative complication (*P*-values < .0001 for all).

## Discussion

As our population continues to age, the incidence of GBM and the proportion of GBM in the elderly continues to increase.^9^ As the rates of these patients entering the operating room increase, we need to learn how to better stratify these patients and improve outcomes in this vulnerable population. The results of this study show that the presence of preoperative medical comorbidities is not associated with decreased survival. Aside from EOR and adjuvant treatment, the presence of a postoperative complication is the most significant predictor of outcome in elderly patients. Postoperative complications are associated with advanced age, longer hospital stay, and discharge destination other than home.

Our study echoes previously published literature demonstrating decreased survival in elderly patients with GBM. The median OS for all patients in our study was 9.4 months (7.8–12.2), compared to 6–12 months in the literature.^5^ In elderly patients receiving palliative radiation only without surgery, the median survival is significantly less with a median survival of 6 months.^12^ Because earlier studies prior to the 2005 Stupp trial tend to report decreased survival in all patients with GBM, we chose 2005 as the starting timeframe for patient inclusion in order to maximize the number of patients receiving TMZ. Similar to previous studies on adult GBM, our study demonstrates survival benefit with STR and GTR compared with biopsy alone, and also survival benefit with GTR compared to STR. While previous studies have found elderly patients undergo less extensive surgery than younger patients,^13^ only 12% of patients in our study had a biopsy alone, compared to 69% that received STR. There were also similar rates of EOR between the two age categories, with patients in the older age category receiving similar surgical care. Upon reviewing the operative notes for these patients, almost all patients received a craniotomy, and surgical debulking occurred after the initial specimen was sent to pathology to confirm the diagnosis.

Our study also found that adjuvant treatment prolonged survival in this population. Treatment with either radiation alone or TMZ alone improved survival compared to no adjuvant treatment, and combined treatment with both radiation and TMZ further improved survival. Unfortunately, few patients received only TMZ and our sample size was too low to directly compare survival between patients that received only radiation or only TMZ. We did find, however, in opposition to EOR, that patients in the older age category were less likely to receive medical care. These patients were less likely to receive any form of adjuvant treatment and more likely to not receive any adjuvant treatment. While our study did not demonstrate survival differences between the two age groups, likely due to being underpowered, because of the adjuvant treatment differences between the age groups our study is not able to directly compare OS between age groups. Previous literature has demonstrated that age as a continuous variable is related to OS, with older patients having worse survival than younger patients.^14^ Similar to EOR in the elderly, previous literature has demonstrated that the rates of adjuvant treatment in the elderly are lower than nonelderly patients.^15^ Recent clinical trials demonstrating survival benefit and no increase in harmful side effects with shortened courses of radiotherapy and combination treatment^7,8^ will hopefully lead practitioners to offer more adjuvant treatment to these patients in the future.

An important finding of our study is that the presence of preoperative medical comorbidities is not related to outcome. A total of 83% of patients in our study had one medical comorbidity, and 55% had multiple comorbidities, with no differences between age groups. This is in keeping with reported literature, which has shown that the number of medical comorbidities increases with age, 75% of patients aged 65 years and older have at least one medical comorbidity, and over 50% of these patients have multiple comorbidities.^16^ Patients with one medical comorbidity or multiple comorbidities had similar survival to patients without medical comorbidities. Diabetes was more common in the older age group, but no single comorbidity was related to survival in either age group. Previous research examining individual medical comorbidities in GBM patients of all ages found that specific comorbidities asthma and hypercholesterolemia were related to decreased survival, however, these comorbidities were present in less than 10% of their study population, and further research confirming these findings is needed.^17^ A recent review of the literature found that the presence of hyperglycemia, rather than diabetes, was an independent risk factor for poor outcome and shorter OS in patients with GBM.^18^ Liu et al. found that lower systolic blood pressure, lower albumin, and higher blood glucose were associated with worse survival.^19^ The driving factor for not only elderly patients but all patients with GBM – and likely those undergoing any type of surgery – is how systemically well these patients are and how well-controlled their comorbidities are perioperative. Elderly patients with multiple well-controlled comorbidities should still be offered surgical and adjuvant treatment for GBM.

Postoperative complications were the most significant predictor of survival aside from EOR and adjuvant treatment. In our study, a total of 30% of patients suffered a medical or surgical postoperative complication. This finding is similar to past research from the Glioma Outcomes Project that found a total of 24% of patients undergoing the first craniotomy for malignant glioma suffered a postoperative complication.^20^ Previous research has also found that postoperative complications are more prevalent in elderly patients and lead to decreased survival in elderly patients with GBM.^4^ In our study, we found that postoperative complications are more likely to occur in patients aged 75 and older, and more likely to be associated with less adjuvant treatment in all elderly patients. We also found patients who suffered a postoperative complication had longer LOS and a discharge destination other than home (either repatriated to a peripheral hospital or discharged to inpatient rehab). While our study does not allow for a causal determination between postoperative complications and LOS or discharge destination, this relationship is likely bidirectional, with immediate postoperative complications including postoperative delirium contributing to a longer LOS, and longer LOS leading to more delayed postoperative complications including pneumonia and UTI. Previous literature has supported both of these cause-and-effect relationships^21,22^ and more recent research also found that much of the variation in LOS is not attributable to patient illness or complications, but instead represented by differences in practice style which can be improved upon through increased efficiency of care and discharge planning.^23^

## Limitations

Our study is a single-center retrospective review and reliant on the completeness and accuracy of the chart documentation. There was also a high proportion of incomplete information regarding tumor mutations and biomarkers, including IDH mutation status and MGMT promoter methylation status. Because the purpose of our paper was to determine clinical predictors of survival, this information was excluded from our study. Finally, our study only included elderly patients undergoing surgery and did not include elderly patients that either refused surgery or were deemed unfit for surgery (palliative).

## Conclusions

Elderly patients account for a large proportion of newly diagnosed GBM and the number of these patients receiving surgical intervention continues to increase. The presence of preoperative medical comorbidities is not a reason to exclude these patients from surgical consideration. Aside from EOR and adjuvant treatment, the most significant clinical predictor of survival in this population during the perioperative period is the presence of a postoperative complication. Patients that are 75 years of age and older are more likely to suffer from postoperative complications, which are associated with a longer LOS and discharge destinations other than home.

## Notes

**Authorship Statement.** M.V. and S.S. performed data collection and drafted the manuscript. M.V. performed data analysis. G.Z. conceived of the manuscript and critically edited and reviewed the manuscript. All authors edited and approved the final manuscript.
